# Development of Alginate Composite Microparticles for Encapsulation of *Bifidobacterium animalis* subsp. *lactis*

**DOI:** 10.3390/gels10110752

**Published:** 2024-11-19

**Authors:** Marko Vinceković, Lana Živković, Elmira Turkeyeva, Botagoz Mutaliyeva, Galiya Madybekova, Suzana Šegota, Nataša Šijaković Vujičić, Anđela Pustak, Tanja Jurkin, Marta Kiš, Sanja Kajić

**Affiliations:** 1 Division of Agroecology, Department of Chemistry, University of Zagreb Faculty of Agriculture, Svetošimunska 25, 10000 Zagreb, Croatia; lzivkovic@agr.hr; 2 Biotechnology Department, M. Auezov South-Kazakhstan University, Tauke-Khan, Shymkent 160000, Kazakhstan; turkeeva_1980@mail.ru (E.T.); mbota@list.ru (B.M.); 3 Chemistry Department, O. Zhanibekov South-Kazakhstan Pedagogical University, Baitursynov Street, 13, Shymkent 160000, Kazakhstan; galiya56@list.ru; 4 Laboratory for Biocolloids and Surface Chemistry, Ruđer Bošković Institute, Bijenička c. 54, 10000 Zagreb, Croatia; ssegota@irb.hr; 5 Division of Organic Chemistry and Biochemistry, Ruđer Bošković Institute, Bijenička c. 54, 10000 Zagreb, Croatia; natasa.sijakovic@irb.hr; 6 Radiation Chemistry and Dosimetry Laboratory, Ruđer Bošković Institute, Bijenička c. 54, 10000 Zagreb, Croatia; apustak@irb.hr (A.P.); tjurkin@irb.hr (T.J.); 7 Faculty of Veterinary Medicine, University of Zagreb, Ul. Vjekoslava Heinzela 55, 10000 Zagreb, Croatia; 8 Division of Agroecology, Department of Microbiology, University of Zagreb Faculty of Agriculture, 10000 Zagreb, Croatia

**Keywords:** alginate composite, agar, casein, *Bifidobacterium animalis* subsp. *lactis* BB-12, encapsulation

## Abstract

The probiotic bacterium *Bifidobacterium animalis* subsp. *lactis* BB-12 (BB-12) was encapsulated in two composites, alginate/agar and alginate/agar/casein. The network structure and physicochemical properties of these composites are influenced by complex interactions, including hydrogen bonding, electrostatic forces between biopolymers, calcium ions, and the encapsulated bacteria. The composites demonstrated a granular surface, with the granules being spatially oriented on the alginate/agar/BB-12 surface and linearly oriented on the alginate/agar/casein/BB-12 surface. They possess a highly organized microparticle structure and exhibit viscoelastic solid-like behavior. The alginate/agar/BB-12 composite showed higher storage modulus, shear stress, and shear strain values, indicating enhanced stability in various physical environments. Both composites displayed good thermal stability, aligning with their rheological properties, confirming their well-ordered structures. Despite differences in composite structures, the release mechanism of bacteria is governed by Fickian diffusion through the composite matrix. Based on physicochemical properties, the alginate/agar/casein composite is recommended for dairy product fermentation, while the alginate/agar composite seems more suitable for oral use. These findings provide new insights into the interactions between bacterial cultures and alginate composite ingredients.

## 1. Introduction

Probiotics, such as *Bifidobacterium animalis* subsp. *lactis* BB-12 (BB-12), are increasingly recognized for their important role in modulating the immune system, regulating the gut microbiota and preventing gastrointestinal diseases [1,2]. However, it remains a challenge to maintain the viability of these beneficial bacteria during food processing, storage and passage through gastrointestinal transit [3,4]. Encapsulation techniques have emerged as viable solutions to protect probiotic bacteria during processing, storage and passage through the gastrointestinal tract [5]. Brown seaweed naturally contains alginate, a polysaccharide commonly used for probiotic encapsulation due to its non-toxicity, biocompatibility and ability to produce hydrogels [6,7]. Its gel formation is usually caused by crosslinking with divalent cations such as calcium, resulting in a network that can efficiently entrap probiotic bacteria [8,9]. Despite its advantages, alginate alone can suffer from limited mechanical strength or a leakage of the encapsulated active ingredients, limiting its protective effect and controlled release ability [10,11]. To alleviate these limitations, blending alginate with other biopolymers has been investigated [12,13]. Blending alginate with polysaccharides or proteins affects physical properties of the composite and improves the ability for targeted applications, especially in the biomedical field and the food industry [14]. For the encapsulation of the probiotic strain BB-12, different mixtures were used to customize the properties of the alginate beads for targeted use [15]. Wang et al. [13] indicated that alginate-based composites (with chitosan, cellulose, starch, proteins, fish gel and other materials) not only increase the survival rates of bifidobacteria but also improve their viability during storage and passage through the gastrointestinal tract. In the current study, we selected a polysaccharide agar and protein, sodium caseinate (CA). Mixing alginate with agar [16,17,18] or casein [19,20,21,22,23,24,25] for probiotic encapsulation can offer several advantages to improve the viability of probiotic bacteria during processing, storage and use, making these composites a promising approach for developing more effective probiotic products. Here are some key benefits. Agar, a polysaccharide derived from red algae, forms strong gels that are less affected by pH changes compared to alginate [16]. Alginate blending with agar offers complementary properties such as high gel strength, stability at higher temperatures, and resistance to enzymatic degradation [17]. The introduction of agar functional groups into the alginate network alters swelling behavior, drug release, mechanical properties, and response to pH [16] and can also improve other properties such as bioactivity, biodegradability, and interaction with cells [18]. Alginate–protein composites have been extensively studied due to their improved mechanical properties, better cell affinity, bioactivity and ability to customize functionality [19]. Casein, the main protein in milk, is an attractive choice due to its high emulsifying capacity, nutritional value and ability to improve the mechanical strength and barrier properties of composite matrices [20,21]. The structural properties of casein and its functionalities make it a very suitable component in food production [22]. In combination with alginate, casein can improve the encapsulation efficiency, the release efficiency of encapsulated bioactive substances in the intestine [21], the structural integrity and the controlled release properties of bioactive substances from microparticles [23,24]. Due to its amphiphilic nature, casein can interact with both hydrophilic and hydrophobic components, which can lead to a more robust encapsulation matrix [25].

Various methods (spray drying, freeze drying, extrusion, emulsification combined with external gelation, ionic gelation, etc.) are used for the encapsulation of BB-12 in alginate-based composites [15,26,27,28,29]. Each method has its advantages and is selected based on the specific application and the desired properties of the encapsulated probiotic [30]. The encapsulation of BB-12 by ionic gelation is a gentle, simple and cost-effective technique that produces microparticles with improved encapsulation efficiency, enhanced viability, and good possibilities of controlled release and stability [31,32]. These properties make ionic gelation a useful technique for the delivery of probiotics in food and pharmaceutical products. This study aims to develop calcium alginate composite microparticles mixed with agar and a mixture of agar and casein to encapsulate BB-12 by ionic gelation. This research focused on evaluating the structural, mechanical and thermal properties as well as the release profile of BB-12 from the microparticles. By utilizing the complementary properties of alginate, agar and casein, the aim is to create an efficient probiotic delivery system with potential applications in the dairy industry and dairy products.

## 2. Results and Discussion

The results are presented and discussed in two parts. In the first part, the interaction between BB-12 cells and calcium ions is analyzed. In the second part, the physicochemical properties of the prepared microparticles are described.

### 2.1. Interaction Between BB-12 Cells and Calcium Ions

#### 2.1.1. Morphology and Size of BB-12 Colony and Cells

**Figure 1:**
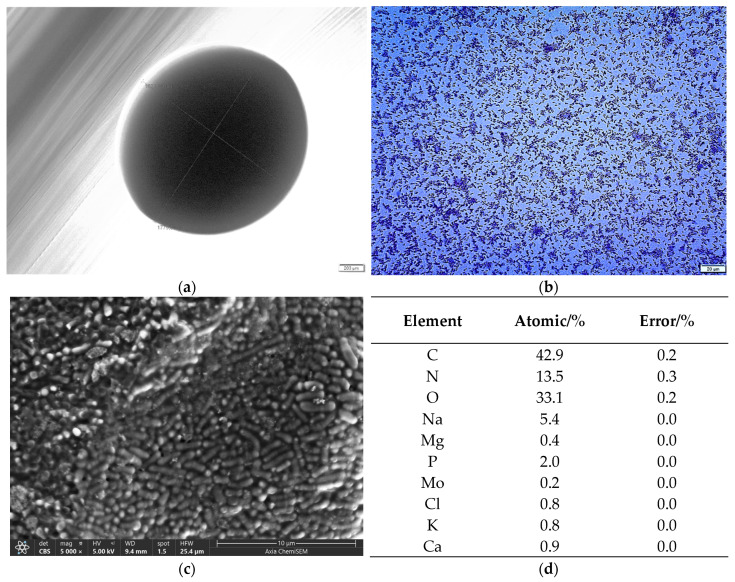
OM microphotographs of (**a**) BB-12 colony and (**b**) Gram-stained BB-12 cells, and (**c**) SEM microphotograph and (**d**) EDS semi-quantitative analysis of BB-12 cells (expressed in the atomic weight percent). The scale bars are indicated on each image.

BB-12 is a type of bacterium characterized by its rod shape and lack of catalase activity. This bacterium belongs to the group of lactic acid-producing, Gram-positive organisms that do not form spores, are non-motile, and thrive in anaerobic conditions [33]. By studying BB-12 cells using different microscopic techniques, the formation of colonies on agar plates consisting of a series of rod-shaped morphology cells was observed. The color of the BB-12 colony is white (Figure 1a). The size of colonies observed by an optical microscope is approximately in the range from 1 to 2 mm, and the length of the rod-shaped cells is approximately from 1.9 to 2.1 µm (Figure 1b,c). These results are consistent with the literature data [34,35]. A semi-quantitative EDS analysis of BB-12 cells revealed the highest percentage of carbon, oxygen, nitrogen and phosphorus (Figure 1d). These elements imply the presence of essential biomolecules such as proteins, nucleic acids and carbohydrates, giving an insight into the structure of BB-12 cells [36]. Other elements were also detected, namely calcium, magnesium, potassium, molybdenum and chlorine.

**Figure 2:**
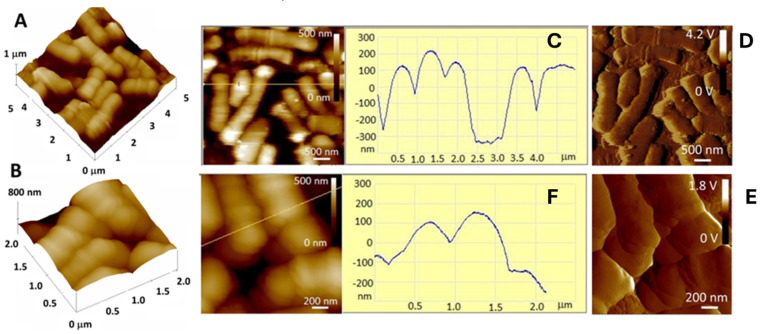
AFM micrographs of BB-12 cells. Visualization of the surface topography and morphology of the cells. (**A**) Topographic image; (**B**) section profile across the marked line; (**C**) amplitude image on a scan area of 5 × 5 μm^2^; (**D**) topographic image; (**E**) section profile across the marked line; (**F**) amplitude image on a scan area of 2 × 2 μm^2^. The scale bars are indicated on each image.

Figure 2 shows the analysis of the surface of BB-12 cells using AFM. According to the topographic images, the BB-12 cells appear to be homogeneously distributed over the mica surface and exhibit cellular integrity with a smooth cell surface structure. The surface remained relatively smooth even after drying during the 1 h measurement. The absence of roughness or irregularities on the cell surface suggest that the cells have no damage, and they are characterized by a well-maintained cell envelope [37]. The height of the cells is 400–500 nm, and the lateral width is about 750 nm.

#### 2.1.2. Effect of Calcium Ion Concentration on the Growth of BB-12 Colonies on Agar Plates and Cells in Suspensions

**Figure 3:**
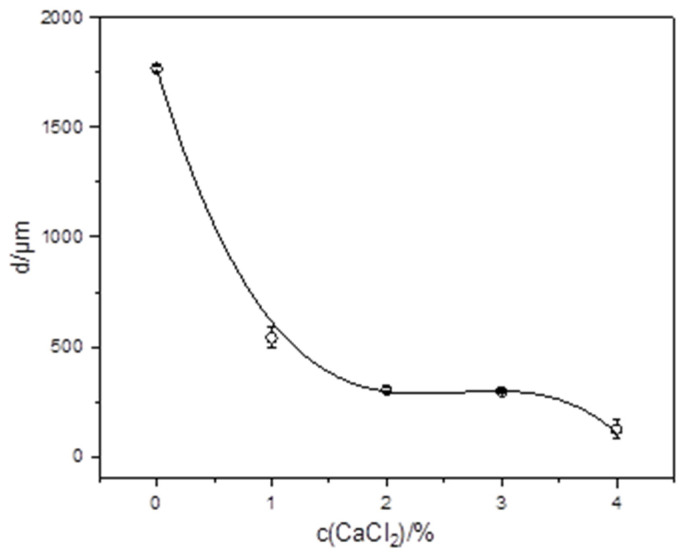
Changes in the mean hydrodynamic diameter (d) and shape of BB-12 colonies with calcium chloride concentration (c(CaCl_2_). Error bars indicate the standard deviation of the means. Inserted microphotographs depict changes in colony shape.

Calcium is essential for the growth and viability of BB-12. It plays a crucial role in maintaining cell membrane integrity and stabilizing cell wall components, which are vital for bacterial growth [38]. By adding a calcium chloride solution (from 1 to 4% *w*/*v*) to agar plates during the growth of BB-12, a change in the size and shape of the colonies was observed. An increase in c(CaCl_2_) leads to a significant decrease in the size of the colonies and loss of their spherical shape (Figure 3). The most pronounced size reduction (up to approximately 10% of the initial value) was observed at the highest concentration of calcium chloride. These changes can be explained by the fact that even low concentrations of calcium chloride can cause a transition from the bifid form to the bacillioid form of BB-12 [39].

**Figure 4:**
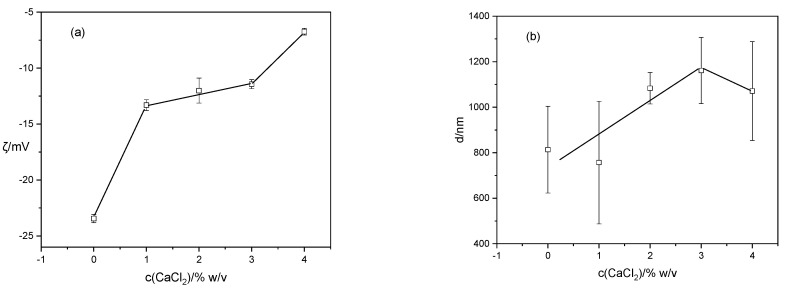
Change in (**a**) zeta potential (ζ) and (**b**) size (d) of BB-12 cell aggregates with increasing CaCl_2_ concentration. Error bars indicate the standard deviation of the means.

Changes in the zeta potential of BB-12 cells and the size of BB-12 cells with increasing concentrations of calcium chloride are shown in Figure 4. BB-12 cells suspended in distilled water are negatively charged with a zeta potential of −23.45 ± 0.37 mV (Figure 4a). The negative potential of cell walls refers to the overall electric charge present on the outer surface of the bacterial cells. This charge is primarily attributed to the presence of various anionic functional groups on the cell surface [40]. Like many other bacteria, the cell wall consists of several layers that serve to protect the cell and maintain its shape. The complex structure of the cell wall of Gram-positive bacteria consists of a thick layer of peptidoglycan with included teichoic acids, polysaccharides and proteins. The outermost layer, often referred to as the “S-layer”, is a proteinaceous lattice that covers the cell surface [41]. Viable or inactive cells bind metal ions to extracellular polysaccharides and cell walls. The accessible functional groups on the cell surface, the kind and concentration of metal ions, surface charge, and metal cations and ligands all affect this process [42]. By adding calcium chloride to the suspension, the zeta potential of BB-12 cells decreased due to calcium binding. Parallel to the decrease in the zeta potential value, the overall particle size increased due to a reduction in repulsion between the cells and their accumulation into aggregates (Figure 4b).

### 2.2. Physicochemical Characterization of Microparticles

#### 2.2.1. Identification of Molecular Interactions Between Microparticle Constituents

**Figure 5:**
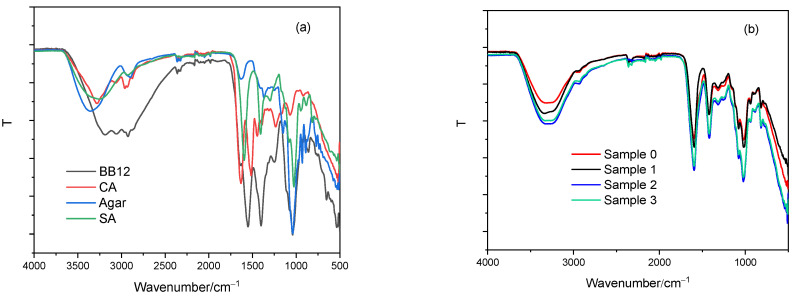
FTIR spectrum of (**a**) single components: BB-12 (black line), CA (red line), agar (blue line), SA (green line), and (**b**) microparticles, and (**b**) ALG/Ca (black line), Sample 1 (red line), Sample 2 (blue line) and Sample 3 (green line).

The spectra of single components (BB-12 cell biomass, agar, CA and SA) are presented in Figure 5a. BB-12 spectrum shows characteristic peaks due to the presence of different cell components that can be divided into five basic areas: fatty acids in the range from 3200 to 2800 cm^−1^, amide bands from proteins and peptides ranging from 1800 to 1500 cm^−1^, mixed regions (proteins and fatty acids) from 1500 to 1200 cm^−1^, polysaccharides within the cell wall from 1200 to 900 cm^−1^, and fingerprints for bacterial cultures from 900 to 500 cm^−1^ as was previously reported [43]. A broad band around 3000 cm^−1^ corresponds to the hydroxyl stretching vibration, and the bands at 2950, 2927, and 2870 cm^−1^ correspond to asymmetric CH_3_ stretching, asymmetric CH_2_ stretching and symmetric CH_3_ stretching, respectively. The small peak at 1648 cm^−1^ is assigned to the carbonyl stretching of amides (amide I) and at 1555 cm^−1^ to the N–H bending of amide II. [44]. The peaks at 1405 and 1300 cm^−1^ are assigned to the bending of −CH_3_ and −CH_2_ groups [45]. The peaks at 1044, 941 and 862 cm^−1^ are detected in the range referring to carbohydrate and phosphate bands [40].

Spectra of both polysaccharides, SA [46] and agar [47,48], as well as protein, CA, [12,49] were previously assigned. The SA spectrum is characterized by broad absorption bands around 3321 cm^−1^ (stretching vibrations of O-H groups) and the stretching vibration of C-H groups at 2920 cm^−1^. Due to the presence of glucuronic and mannuronic acids, intensive stretching peaks related to carboxylate groups (COO^-^) at 1595 cm^−1^ (asymmetric) and 1405 cm^−1^ (symmetric) can be seen. Bands found within the range of 900 to 1200 cm^−1^ signify a polysaccharide structure and strong peak at 1026 cm^−1^ stretching vibrations of C-O-C groups. The spectrum of agar shows a broad band around 3440 cm^−1^ (free, inter- and intra-molecular hydroxyl groups), C-H stretching vibrations at 2920 cm^−1^ attributed to methoxyl groups, a peak at 1630 cm^−1^ attributed to the stretching vibration of the conjugated peptide bond formation by amine (NH) and acetone (CO) groups, and peaks at 1370 cm^−1^ associated with an ester sulfate group and at 1150 cm^−1^ associated with glycosidic linkage vibrations. Bands at 1041 and 921 cm^−1^ represent the C-O stretching group of 3, 6-anhydro-galactose and the peak appearing at 887 cm^−1^ is associated with the C-H stretching of residual carbons of β-galactose. The sodium caseinate spectrum shows an absorption maximum at 3292 cm^−1^ assigned to the O-H stretching vibrations overlapped with the N-H stretching vibrations, a small band extending from 3100 to 2850 cm^−1^ with a peak at 2954 cm^−1^ related to the C-H groups, and intense peaks at 1630 cm^−1^ and 1520 cm^−1^ related to amide groups (amide I and amide II, respectively). The peak at 1420 cm^−1^ corresponds to O–C–O groups and the peak at 1231 cm^−1^ to the C-C(O)-C stretching vibrations of esters present in sodium caseinate. The peak at 1053 cm^−1^ may be a contribution of different groups such as C-H and PO^2−^ or P–OH bending vibrations [50]. Spectra of control microparticles and composites loaded with BB-12 spectra are presented in Figure 5b. In relation to SA, changes in the spectrum of Sample 0, caused by the replacement of sodium ions with calcium ions of a larger ionic radius, are visible in the area of functional groups where hydroxyls and carboxylates are located. The characteristic bands of Sample 0 are identified by the hydroxyl stretching vibration occurring around 3300 cm^−1^, peaks associated with carboxylate groups (COO^−^), asymmetric stretching vibrations at 1589 cm^−1^ and symmetric stretching at 1413 cm^−1^, along with peak stretching vibrations of C-O-C groups at 1024 cm^−1^ as was shown in our previous work [51]. Spectra of composites show no distinct agar or CA vibrations indicating their involvement in the calcium alginate gel network. Composites loaded with BB-12 show the absence of BB-12 characteristic bands confirming successful encapsulation. The main bands of alginate dominate in all composites. All spectra of composites revealed higher intensity and wider absorption bands associated with stretching vibrations of O-H groups as well as the shifting of peaks associated with COO^−^ groups (asymmetric and symmetric) and C-O-C groups indicating an increase in hydrogen bonding and electrostatic interactions compared to calcium alginate. Hydrogen bonding plays an important role in the structure and properties of alginate hydrogels influencing the crosslinking density [52]. Both stronger hydrogen bonds and electrostatic interactions contribute to a higher crosslinking density of composites loaded with BB-12.

The complex molecular interactions between the composite ingredients are governed by the nature of their functional groups. Some interactions may already occur before the crosslinking process. This includes the complexation of alginate carboxyl and hydroxyl groups with CA amide groups [12], and hydrogen bonds are formed between two polysaccharides, alginate (hydroxyl and carboxylate) and agar (hydroxyl and sulfate) [53,54]. CA functional groups (carboxyl, amino and phosphate) can participate in hydrogen bonding, ionic interactions and hydrophobic interaction [55]. The presence of BB-12 functional groups also contributes to the overall interactions between components in the mixtures. By adding biopolymers and BB-12 to the crosslinking solution, in addition to the crosslinking of alginate with calcium ions [56], the interactions of calcium ions with other components are also possible. Agar can undergo gelation in the presence of calcium ions due to the binding of Ca^2+^ [57] and CA above its isoelectric point (from 4.6 to 4.9 [58]) displays a negative charge that can form ionic bonds with calcium ions. The binding of calcium ions to BB-12 (Figure 4a) also affects the concentration of calcium available for alginate crosslinking. Complex molecular interactions collectively contribute to the structure of the microparticles.

#### 2.2.2. Microscopic Characterization of Microparticles

The OM observations showed that wet control microparticles were transparent but those loaded with BB-12 were white and egg-shaped. Composites containing CA became slightly elongated. This elongation can be explained by the viscosity change when water is replaced by the casein in the preparation process [14]. In addition to calcium chloride concentration and alginate properties (the molecular weight and proportion of G-blocks), the solution flow rate and the distance between the point from the nozzle to the gelling bath, the size of the nozzle is very important [59]. The approximate size of the microparticles was prepared under our experimental conditions, ranging from 1284 ± 73 µm (Sample 0), 1482 ± 162 µm (Sample 1), 1852 ± 82 µm (Sample 2) and 2185 ± 172 µm (Sample 3), respectively.

**Figure 6:**
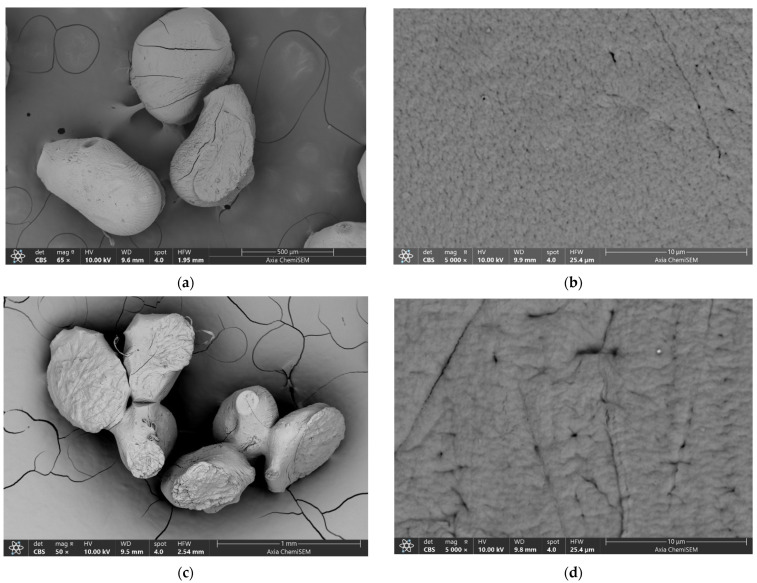

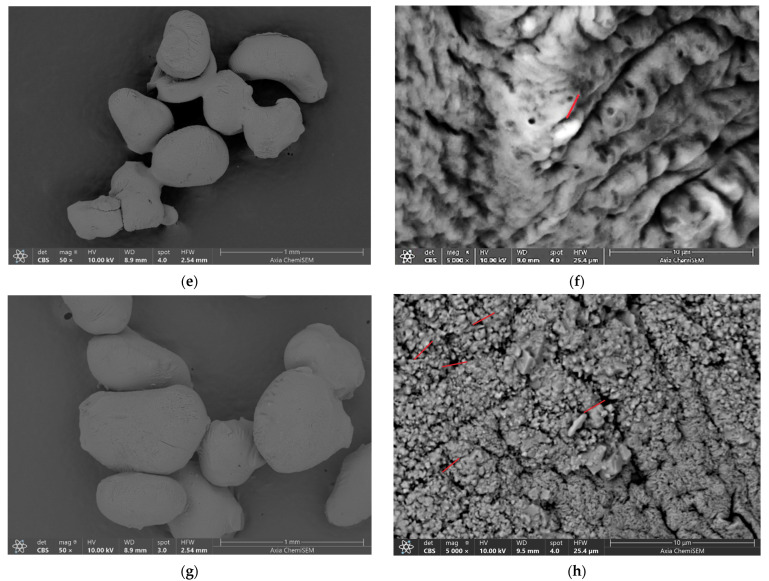
SEM microphotographs of Sample 0 (**a**,**b**), Sample 1 (**c**,**d**), Sample 2 (**e**,**f**) and Sample 3 (**g**,**h**). BB-12 cells located on the surface of microparticles are denoted by red lines. The scale bars are indicated on each image.

Figure 6 shows SEM microphotographs at different magnifications. After drying (on air at room temperature) to a constant mass, all microparticles became smaller (on average by about 52% (Sample 0), 41% (Sample 1), 32% (Sample 2) and 33% (Sample 3), and more or less deformed (Figure 6a,c,g,h). During drying, water is removed from the microparticles, causing them to shrink. The surface of the microparticles was porous with wrinkles and cracks that appeared as a result of the stress relaxation process of the biopolymers associated with the loss of water and moisture [60]. Compared to the surface of Sample 0, composites revealed changes in morphology and porosity. The Sample 0 surface shows an average pore size of 160 nm (Figure 6b), which is consistent with our previous work [61]. The smallest average pore size (144 nm) can be seen on the surface of Sample 1 (Figure 6d), and the largest (563 nm) on the surface of Sample 2 (Figure 6f). Sample 3 shows an average pore size of 150 nm (Figure 6h). Enlarged microphotographs (Figure 6f,h) reveal rod-like structures (denoted by red lines) indicating the penetration of BB-12 cells through the surface, as has been similarly observed for Lactobacillus sakei [42]. The higher number of BB-12 cells on the surface of Sample 3 may be attributed to slightly faster diffusion within the microparticle matrix compared to Sample 2. EDS semi-quantitative analyses of the area nearest to the surface (the electron probe can penetrate to a depth of about 1 μm) showed that the major percentage of elements in all microparticles corresponds to C, O and Ca. A small amount of detected Na and Cl were probably residues of compounds used during the preparation.

**Figure 7:**
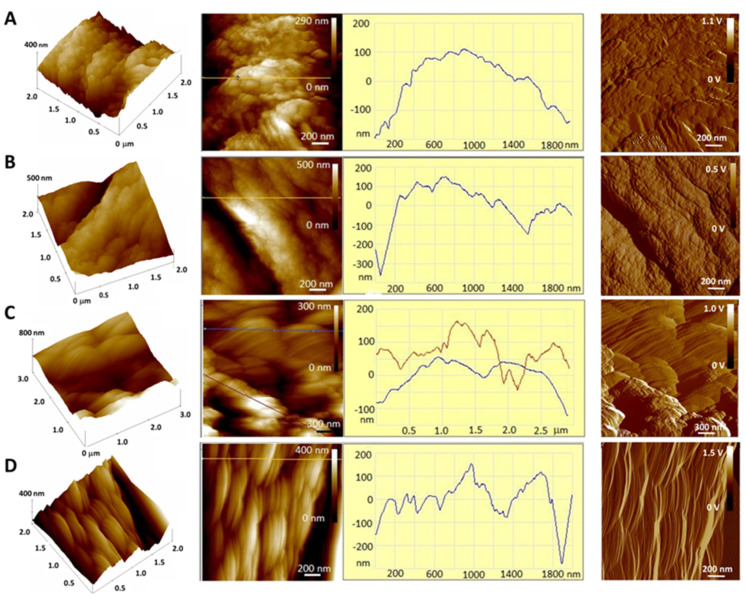
AFM micrographs of microparticles Sample 0 (**A**), Sample 1 (**B**), Sample 2 (**C**) and Sample 3 (**D**) with 3D topographic images of height data (**top** view), section analysis profiles (**right**) along labeled lines (**left**) and amplitude images (**top** view) of microparticles. The scale bars are indicated on each image.

The surface morphology of the prepared microparticles revealed by AFM is shown in Figure 7, in which the scanned microparticle surface represented by topographic images of the height data is shown as a “top view” characterizing the morphology of each microparticle of different compositions and as a “3D top view” with a corresponding color scale showing the 3D height distribution on the microparticle surface. In addition, the characteristic section profile (“section analysis”) of a single microparticle shows an analyzed 2D height distribution. Samples 0 and 2 have granules on the surface that are not spatially oriented, i.e., they are linearly organized in different directions and maintain their shape, with a height and lateral spacing in the range of 50–100 nm and 250–400 nm, respectively. In contrast, Sample 1 and Sample 3 have regular, elongated, ellipsoidal granules on the surface (with a height and lateral dimension of 80–90 nm and 200 nm, respectively), which are linearly oriented. In our previous work, we observed a similar oriented structure [62]. Compared to Sample 0, the addition of agar in Sample 2 shows a decrease in the roughness value due to its placement in the interstitial space, which simultaneously reduces the roughness value to 59 ± 3 nm (Table 1). On the other hand, the addition of casein leads to an increase in roughness values in Samples 1 and 3, with linear orientations dominating on the surface (see Figure 7).

#### 2.2.3. Rheological Properties

##### Amplitude Sweep

**Figure 8:**
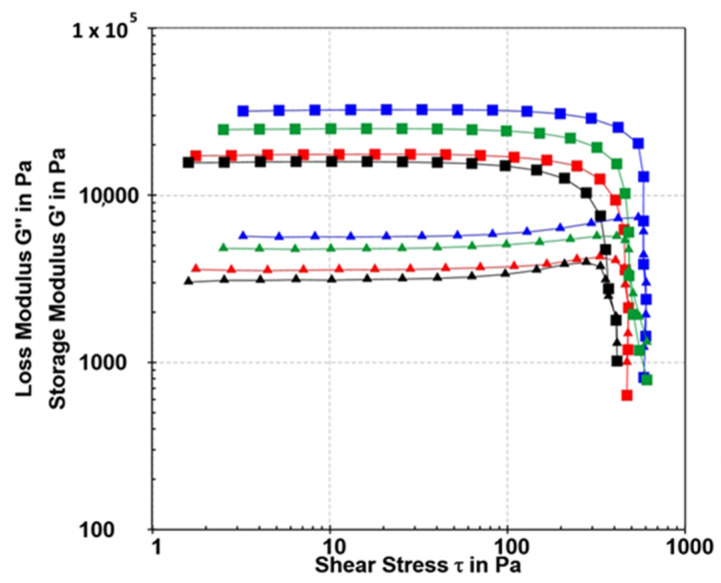
Amplitude sweep tests (G′ (■) and G″ (▲) values) of Sample 0 (red), Sample 1 (black), Sample 2 (blue) and Sample 3 (green) determined at a constant angular frequency of 5 rad/s at 23 °C.

The rheological properties of alginate gels provide important information about their strength and stability during processing and application [63,64]. The viscoelastic properties of the microparticles were analyzed using oscillatory rheology. The storage modulus G′ (Pa) quantifies the elastic portion of viscoelastic behavior, representing the sample’s solid-state characteristics. Generally, the elastic modulus (G′) is higher than the loss modulus or viscous component (G″) at low applied shear, achieving a plateau within the linear viscoelastic region (LVR). This region is defined as the range where the applied stress has little effect on the material’s three-dimensional structure. The yield point marks the disruption of the linear stress–strain relationship, indicating structural changes in the polymer. In the amplitude test, the frequency remains constant while the shear strain varies. Strain (γ) sweep tests were conducted in the strain range from γ = 0.01 to 100% at an angular frequency ω = 5 rad/s at 23 °C. The G′ and G″ values remained nearly unaffected by the applied strain up to 0.5%. The amplitude sweep results are presented in Figure 8.

The amplitude sweep results reveal two sample profiles, one for the control samples and the other for composites loaded with BB-12. The control samples exhibit similar values. The storage modulus (G′) for Sample 0 is 17,524 Pa, while for Sample 1 with casein, it is slightly lower at 15,942 Pa (Table 2). Besides the slightly lower storage modulus, other values for the sample with casein are also lower compared to calcium alginate microparticles. The yield point for calcium alginate is 240.1 Pa, whereas for the calcium alginate/casein composite, it is 173.9 Pa. The flow transition index is somewhat higher in the case of the alginate/casein composite. Casein affects the microstructure of the calcium alginate through interactions of casein amide groups with alginate hydroxyl and carboxyl groups. At a predominant concentration of alginate, a transition of the structure into a bicontinuous phase was observed, where both alginate and casein formed interconnected networks [65]. The flow transition index represents a measure of the yielding zone, a valley between a yield point and a flow point. The higher value of the flow transition index of Sample 1 compared to Sample 0 indicated higher resistance to applied stress [54]. Composites loaded with BB-12 (Sample 2 and Sample 3) show a change in viscoelastic properties. The addition of agar to alginate increases the strength of Sample 2, and the storage modulus is 35,700 Pa. However, the addition of casein to Sample 3 reduces the strength to 25,052 Pa. The yield point values for the samples with agar are significantly higher than those of the control samples, indicating a higher crosslinking density. For Sample 2, the yield point is 377.8 Pa, while for the Sample 3, it is 281.9 Pa. It is shown again that casein affects the viscoelastic properties of the composite. However, despite forming somewhat softer microparticles, the viscoelastic properties of samples prepared with casein are maintained. All prepared samples possess a highly organized structure, with loss factor values ranging from 0.17 to 0.21. The quantity loss factor, tan(δ) = G″/G′, determines the relative elasticity of viscoelastic materials.

Studies have shown that the viscoelastic properties of alginate gels are closely linked to their microstructure [66]. The arrangement and interaction of alginate chains within the gel determine the degree of crosslinking and the distribution of crosslinking sites within [67]. A higher crosslinking density makes the gel more resistant to deformation and maintains its structure under applied stress, resulting in a higher yield point [54]. The lower loss factor values for Sample 2 and Sample 3 compared to the control samples can be attributed to the higher crosslinking density. Gels with a higher crosslinking density are usually stiffer and less deformable and have lower energy dissipation (lower loss factor) [52]. Also, higher values of storage modulus, yield strength and yield point for Sample 2 and Sample 3 are associated with a higher degree of crosslinking, indicating a tighter network structure with increased mechanical strength [54]. However, it is noteworthy that despite the variation in structure with the addition of agar, BB-12 or casein to calcium alginate, all investigated samples demonstrated high elastic modulus (G′) values in the order of 104 Pa. Blending alginate and agar (Sample 2) can form a dual-network structure, which can improve the strength and elasticity of the material [68]. This makes it suitable for applications that require robust and flexible gels. Alginate blending with agar and casein creates a composite gel with a complex, multi-phase structure. The alginate and casein form interpenetrating networks, while the agar adds structural support and stability [54].

##### Frequency Sweep

**Figure 9:**
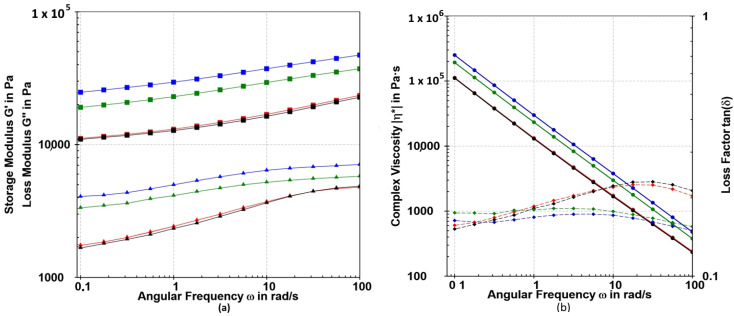
(**a**) Frequency sweep test (G′ (■) and G″ (▲) values) and (**b**) complex viscosity (ƞ*) and loss factor (tan δ) of Sample 0 (red), Sample 1 (black), Sample 2 (blue) and Sample 3 (green) determined at a strain of 0.1% at 23 °C.

Frequency sweep analysis offers a means of characterizing the time-dependent response of samples under non-destructive deformation, shedding light on the behavior and internal structure of polymers. In this study, frequency sweep tests were performed over a range of 0.1–100 rad/s at 0.1% strain within the linear viscoelastic region (LVR) at 23 °C. The results revealed that, across the entire frequency spectrum, the storage modulus (G′) consistently exceeded the loss modulus (G″) (G′ > G″), indicating a predominantly elastic rather than viscous nature for the samples, as illustrated in Figure 9. The frequency-dependent progression of both G′ and G″ was distinct for the samples, displaying two characteristic profiles: one for Sample 0 and Sample 1 and another for Sample 2 and Sample 3. Notably, the highest G′ values were observed in composites containing BB-12. At elevated frequencies, the samples exhibited greater rigidity, as reflected in the increased G′ values (Figure 9a). The gradual increase in the elastic modulus with frequency suggests relaxation processes, potentially due to the release of reversible entanglements or the opening of intermolecular junctions [69]. Generally, the elastic modulus of the alginate polymer is influenced by the number of crosslinks, as well as the length and stiffness of the chains between these crosslinks [70]. It is considered that a low angular frequency (ω) region is considerably sensitive to a polymer network structural change. Shear-thinning behavior was observed in all examined samples, as the complex viscosity decreased with increased frequency (Figure 9b). The loss factor for control samples changed from 0.15 at lower frequencies to 0.22 at higher frequencies.

The frequency sweep for the composites loaded with BB-12 showed a different profile with less frequency dependence compared with the control samples. The loss factor remained in the range of 0.16–0.17 throughout the entire frequency range for the composites loaded with BB-12. However, all examined samples exhibited similar values around 0.16 at frequencies lower than 1 rad/s. At higher frequencies, composites loaded with BB-12 showed less frequency sensitivity, indicating higher structural ordering, in contrast to the control samples. The composites loaded with BB-12 demonstrated consistency throughout the entire examined range, indicating stability for a prolonged period of time.

##### Creep and Creep Recovery Test

**Figure 10:**
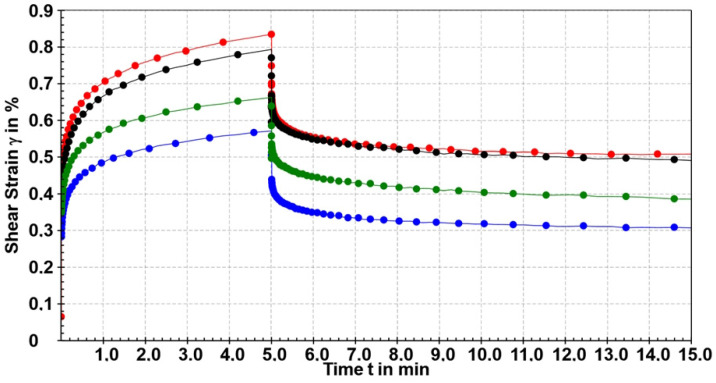
Time-dependent strain variation during both the creep and creep recovery tests for Sample 0 (red), Sample 1 (black), Sample 2 (blue) and Sample 3 (green) at 23 °C.

The influence of casein, agar and BB-12 in microparticles can also be assessed for their impact on the internal structure of calcium alginate through creep and recovery studies (Figure 10). Creep and recovery tests involve subjecting a viscoelastic material to deformation for a specific duration under constant shear stress within the linear viscoelastic region, measuring the deformation per unit of stress (compliance J) over time (creep test). Subsequently, the applied stress is removed, and the deformation over a specific period is measured (recovery step). Recovery profiles were determined by plotting the compliance (J) as a function of time. To investigate the relationship between the microstructure and rheological properties of the microparticles, their creep profile was fitted to the Burgers model (Equation (4)) and their recovery profile to Equation (5) [71]. The creep represents a slow and progressive deformation of the material under constant stress. The creep function describing the time-dependent deformation behavior during the stress phase can be formulated as follows:

(1) $$\gamma \left(t\right)=\frac{\tau_{0}}{G_{1}}+\frac{\tau_{0}}{G_{2}}\cdot [1-exp(-t/\Lambda )]+\frac{\tau_{0}\cdot t}{\eta_{0}}$$

Shear modulus *G*_1_ represents instantaneous elastic modulus and defines the samples’ resistance to deformation (i.e., the immediate deformation step due to purely elastic behavior instantaneously recovered when the stress is removed); the retardation time (*Λ*(*s*) = η2G2) is a measure for the delay in the material’s full elastic response to an applied stress, caused by its viscous element; and *G*_2_ represents the contribution of the retarded elastic region to the total compliance. Zero shear viscosity (*η*_0_) is determined at the end of the creep phase, when steady-state flow behavior is reached (i.e., dγdt = const.).

The creep compliance function, describing the time-dependent reformation behavior during the rest phase, can be formulated as follows:

(2) $$J\left(t\right)=J_{max}-J_{0}-\sum J_{m,i}\cdot (1-\exp\left(\frac{-\left(t-t_{0}\right)}{\Lambda_{i}}\right)+(\frac{t-t_{0}}{\tau_{0}})\cdot \left(\frac{d\gamma }{dt}\right))$$

Creep compliance (*J*(*t*)) can be calculated using the preset shear stress *τ*_0_ and the resulting deformation function γ(*t*) obtained in the creep phase. Instantaneous compliance (*J*_0_) is the limiting value of the *J*(*t*) function at the very beginning of the creep test (*t* = 0) and $\gamma_{max}$ represents the maximum deformation reached at the end of the creep stage. *J_max_* is the maximum compliance achieved during the creep profile. Viscoelastic compliance *J_m_*_,*i*_ represents a slow or retarded recovery due to a decreasing exponential type and tends toward an asymptote when t→∞. Compliance *J_e_*_0_ represented in Table 3 is the total elastic compliance, also shown as elastic compliance *J_e_* in %.

The stress applied for characterizing the creep profile and recovery of the tested samples was determined based on the results of the amplitude sweep and critical stress values. Creep and recovery studies were conducted under a constant stress of 30 Pa for a duration of 5 min. The creep and recovery profiles show the same general behavior (Figure 10). During the creep phase, all samples demonstrate progressive polymer network deformation under constant stress. Upon stress release, the samples exhibited immediate recovery followed by a gradual reduction in deformation until reaching a constant, non-zero value. The results obtained from the creep study can be compared with the amplitude sweep experiments. It was found that Sample 0 was the most sensitive to deformation under constant stress, reaching a maximum strain of 0.83%, which was at the limit of the linear viscoelastic region, while a comparable maximum strain of 0.80% was measured for Sample 1 (Table 3). The most resistant structure to deformation under constant stress was Sample 2 with a maximum deformation of 0.57%, while Sample 3 exhibited a slightly higher deformation of 0.66%, which was significantly less than that of Sample 0.

As compliance (J) is inversely related to the storage modulus (G), J = 1/G, higher G_0_ results in lower J_0_, indicating greater resistance to strain. J_max_, representing the maximum compliance during the creep profile, shows an inverse relationship with G′ and G_0_. The values G′ and G_0_ are directly associated with the network’s resistance to deformation (i.e., the higher J_max_, the lower the polymer resistance to deformation).

Sample 0 and Sample 1 showed the lowest resistance to instantaneous deformation (J_0_), followed in increasing order by Sample 3 and Sample 2 (Table 3). Elastic compliance evaluated in percentages is 35.3% for Sample 0 and a slightly lower value of 32.4% for Sample 1. The influence of casein was observed again as it led to a decrease in the elastic compliance in Sample 3 to 36.2% compared to 41.9% in Sample 2. Since the elastic modulus of polymer networks is proportional to the number density of crosslinks, the decrease in the initial modulus with the addition of casein suggests a significant role in network formation. The viscous behavior towards the end of the experiment is interpreted as a balance between bond breaking and formation. The Kelvin elements (J_m,i_) described different interactions with characteristic retardation times (*Λ*) in the range of 1 s and 62 s. Assigning specific interactions is complex, and understanding these interactions at a molecular level would facilitate a more rational search for optimal delivery materials.

#### 2.2.4. Thermal Properties

**Figure 11:**
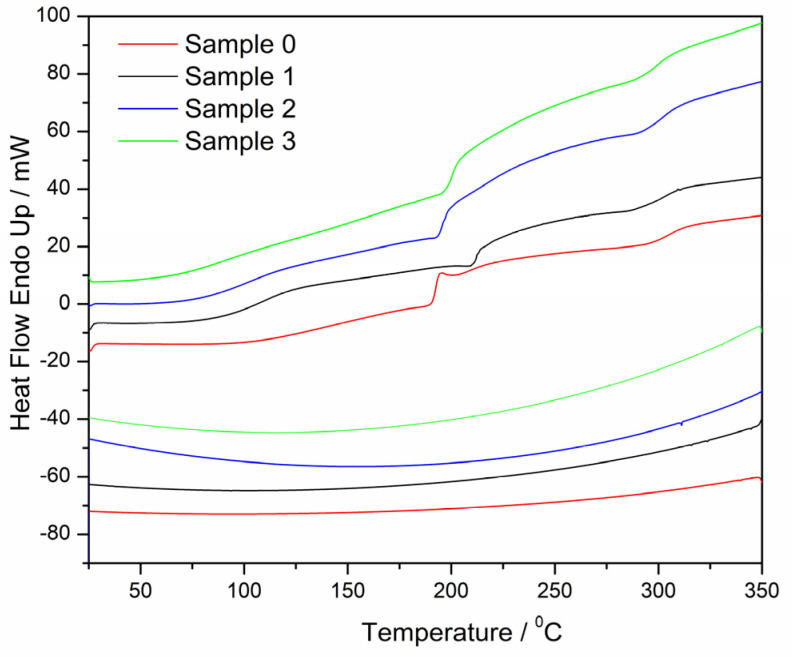
DSC curves of Sample 0 (red line), Sample 1 (black line), Sample 2 (blue line) and Sample 3 (green line) at a heating rate of 10°/min (the first heating cycle and cooling cycle).

The investigation of the thermal stability of biopolymers and their formulation in different forms, e.g., alginate microparticles, is very important for possible storage and other applications. Also, the release rate of active components is strongly dependent, among other things, on temperature [72]. The thermal properties of microparticles were investigated by DSC analysis, the common thermal analysis for this type of polymer. Thermograms of the first heating and cooling cycle are presented in Figure 11.

There are no thermal changes in the cooling cycle, which implicated mostly the amorphous nature of polymers and their gel structure. All transition temperatures and enthalpies are calculated from the first heating cycle at a heating rate of 10 °C/min and presented in Table 4. All the glass transition temperatures (relaxation processes) are determined from the inflection point method and the represented temperatures and enthalpies are calculated with Pyris Software(Version 11) for Perkin Elmer DSC and normalized before analysis. As in the previous investigation [73,74], the thermograms in the heating cycle show three distinctive transition peaks for all samples. A very broad endothermic change in a temperature range of 80–120 °C pointed towards the loss of water. Alginate hydrogels contain pools of water characterized as a free water phase of high mobility (occupying macropores) and a bound water phase of limited mobility (connected to the network structure) [75]. Very important for usage in drug delivery or possible storage is a fact that the composite microparticles are stable until 80–100 °C [76]. The enthalpies are not calculated since the peaks are very broad, and it is difficult to determine the beginning and the end of this transition, so only temperatures are presented. A very distinctive transition between 190 and 210 °C with a noticeable change in heating capacity C_p_ in the heating curves of all samples can be assigned to a glass transition change, or a kind of relaxation process in the polymer, and is probably attributed to the calcium alginate gel network transition. This change is followed by an exothermic peak that is possibly due to cold crystallization following the relaxation process before final polymer degradation occurs, including saccharide ring depolymerization and polymer decomposition, as reported in previous investigations [73,74]. It is a type of exothermic anomaly caused by changes in the polymer after reaching the viscoelastic state and is observed for all samples [77,78]. The relatively high temperature of this transition can lead to the conclusion that the further thermal stability of the microparticles is achieved before they undergo further structural changes by heating. The exothermic change around 290 °C occurs due to a change in the orientation of the chains or polymer networks due to the softening of the material after the relaxation process (it goes into a viscoelastic state before degradation) [79]. The polymer chains can be oriented to form crystalline domains to achieve recrystallization that is then seen on DSC as an exothermic change.

The degree of crosslinking in a gel affects its thermal properties. A higher crosslinking density limits the molecular motion, thus altering the energy required for phase transtions [52]. The degree of crosslinking in alginate can significantly influence its glass transition temperature. Compared to calcium alginate (Sample 0), the increase in T_g_ (Table 4) for the composite microparticles indicates an increase in crosslinking density. The higher crystallization enthalpy change (ΔH_cc_) observed for the control samples compared to the BB-12-loaded composites indicates reduced energy for phase transition. The correlation between the crystallization enthalpy change and crosslinking of alginate refers to the heat released when a substance changes from a liquid to a crystalline solid. The change in the enthalpy of crystallization (ΔH_cc_) of Sample 2 and Sample 3 (Table 4) is consistent with higher values of storage modulus (Table 2) compared to the control samples.

#### 2.2.5. BB-12 In Vitro Release from Composites

**Figure 12:**
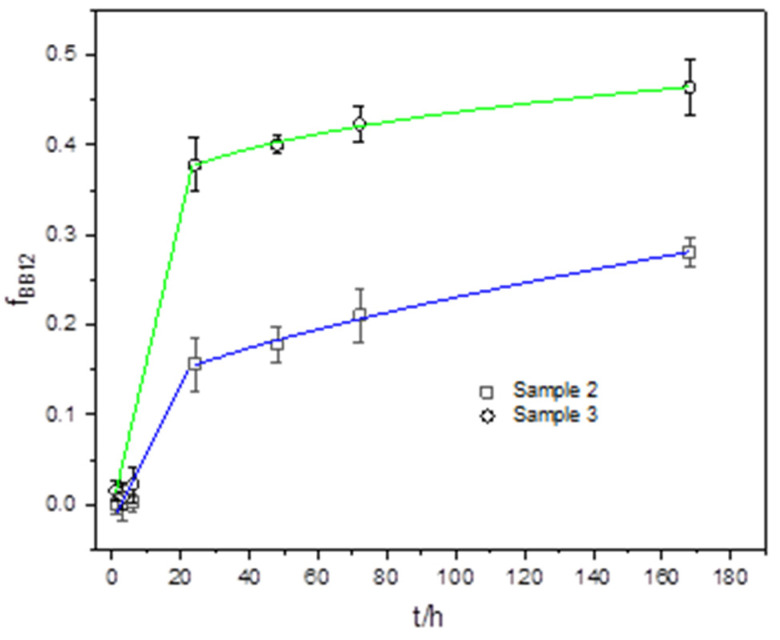
Fraction of released BB-12 cells (f_BB-12_) from composites with time (t). Error bars indicate the standard deviation of the means. Samples are denoted.

One of the most important properties of alginate gel is the possibility to control the release of encapsulated ingredients. The main processes governing release from hydrophilic polymer microparticles are swelling, dissolution/erosion at the matrix surface, and diffusion through the matrix [80]. The release profiles of BB-12 from Samples 2 and 3 dispersed in water, shown in Figure 12 as changes in the cumulative fraction of BB-12 released over time, reveal the initial release of a negligible amount of BB-12 followed by an abrupt release and a slower release interval. The initial time is equivalent to the time required for the microparticle to hydrate and reach equilibrium before the BB-12 release begins. The processes involved during this phase are the penetration of water and the filling of surface pores of microparticles with water. Following the burst phase, the release tends to slow down, entering a phase of sustained release governed by diffusion through the gel matrix. Release profiles are analyzed by a modified semiempirical Korsmeyer model [81] and presented as fraction of cumulatively released BB-12 by the following equation [82]:

f_BB-12_ = a + kt^n^, (3)

where t is the release time, k is a kinetic constant characteristic for a particular system considering structural and geometrical aspects, n is the release exponent indicative of the BB-12 rate controlling mechanism (n < 0.43 (diffusion), 0.43 < n < 0.85 (a combination of diffusion and polymer swelling and relaxation), and n > 0.85 (polymer swelling and relaxation)), and a is the *y*-axis intercept. The release exponent values less than 0.45 (n = 0.27 and n = 0.36 for Sample 2 and Sample 3, respectively) revealed that the rate-controlling release mechanism is Fickian diffusion. Sample 3 revealed a higher amount of BB-12 released at a somewhat higher rate (k/h = 0.52 and k/h = 0.63 for Sample 2 and Sample 3, respectively).

Alginate crosslinked with Ca^2+^ forms a three-dimensional network with channels for the transport of encapsulated ingredients. When calcium alginate is blended with agar or agar and CA, the number of alginate strands held together in the three-dimensional network changes due to molecular interactions between functional groups before and during crosslinking. When mixed with alginate, the agar network induces a transformation from a distinct layered structure to a co-continuous network followed by a crosslinking density increase, which slows down the release of encapsulated bacteria [68]. The incorporation of sodium caseinate into Sample 3 has a further effect on the structure compared to the Sample 2 network. Casein contains phosphate groups that can chelate calcium ions [54] and thus reduce the availability of free calcium for the formation of crosslinks in the alginate matrix. The microparticles become softer with a structure that captures bacteria less efficiently (EE = 39%, LC = 1.3 × 10^5^ CFU/g) compared to Sample 2 (EE = 52%, LC = 1.5 × 10^5^ CFU/g).

The degree of swelling is related to the crosslinking that occurs during the gelation process between the calcium ion and the alginate chains, and therefore, S_w_ can be considered as measure of the extent of crosslinking [42]. The crosslinking degree for Sample 3 was higher (S_w_ ≅ 53%) than for the Sample 2 (S_w_ ≅ 46%), indicating a lower crosslinking density, less dense network structure and higher amount of absorbed water. A high ratio of free water with high mobility is useful for the diffusion of encapsulated ingredients, ensuring the survival, migration and proliferation of the encapsulated cells [75]. Despite the larger pores of Sample 2, Sample 3 releases BB-12 faster and more of it. This can be attributed to the less dense structure and softer microparticles of Sample 3 and fewer calcium ions available to bind to BB-12, thus facilitating bacterial diffusion through the matrix. The higher amount of BB-12 found on the surface of Sample 3 (Figure 6h) than on the surface of Sample 2 (Figure 6h) also appears to have contributed to the higher amount of BB-12 released.

In general, it is desirable that the microparticles delivering the probiotic to the colon withstands the acidic environment of the stomach and enzymatic activity in the small intestine, ensuring that a significant number of viable bacteria reach the colon. In addition to the slower BB-12 release, the advantage of adding agar to alginate microparticles is greater stability at different pH values [16]. On the other hand, the addition of casein releases a larger amount of BB-12 in a shorter time, giving it an advantage for use in various fermentation processes. By using these two complex matrices, it is possible to tailor the release by extending the time the bacteria remain encapsulated, which is an advantage for applications that require sustained release. 

## 3. Concluding Remarks

Composites loaded with *Bifidobacterium animalis* subsp. *lactis* BB-12 (alginate/agar/BB-12 and alginate/agar/casein/BB-12) and the control samples (calcium alginate and composite calcium alginate/casein) were prepared by ionic gelation. The presented work shows how important the choice of materials is to obtain the customized structures required for the encapsulation of probiotic bacteria for targeted use, as each component of the microparticles contributes to the overall complexity. Molecular interactions (mainly hydrogen bonds and electrostatic interactions) between biopolymers and interactions with calcium ions, as well as the presence of encapsulated bacteria, have a complex effect on the network structure and surface morphology of microparticles.

There is a correlation between the microstructure of the samples and the storage modulus. It is important to note that the influence of casein on the microstructure is visible in the control sample (Sample 1) as well as in the composite with agar (Sample 3). In both cases, but in different ways, the impact of casein on the surface texture of the material is observed, along with an increase in roughness, which correlates with the decrease in the storage modulus values for the tested samples. We would like to point out that the storage moduli for all four samples are on the order of 10^4^ Pa, which describes solid samples, and their loss factors, which indicate their structural organization, are similar for both agar-free and agar-containing alginate samples. The most significant effect on the change in storage modulus relative to alginate microparticles is observed with the addition of agar, which also influenced the change in microstructure visible in SEM and AFM micrographs. Another important factor is the impact of casein on the yield point in both the alginate control sample and the samples with added agar. Based on the storage modulus, yield point, and duration of the softening of the material, it can be concluded that the microstructure of casein samples is more susceptible to deformation, which can be correlated with microscopic images. However, the most noticeable effect is seen in the alginate/agar/casein composite, where the structure changes completely, resulting in a porous structure that contains smaller particles, has a large surface area, and, consequently, a much faster release of bacteria compared to the alginate/agar composite. The influence of agar and casein on morphology is also evident in DSC in the first observed transition, where an earlier change is detected in the alginate/agar composite than in the alginate/agar/casein composite. Regarding practical applications, the thermal treatment of composites with agar or with both agar and casein up to 85–90 °C would not affect the properties of the products.

The release profiles of the probiotic bacteria showed that diffusion is a rate-controlling mechanism for both composites. The higher release of BB-12 from the alginate/agar/casein/BB-12 composite can be attributed to the lower crosslinking density, slightly softer microparticles and less calcium available to bind to BB-12, which facilitates the diffusion of bacteria through the matrix. Considering the release behavior as well as the mechanical and thermal properties, the alginate/agar/casein composite can be recommended in dairy fermentation, while the alginate/agar/casein composite seems more suitable for oral use.

## 4. Materials and Methods

### 4.1. Materials

Sigma Aldrich (St. Louis, MO, USA) supplied alginic acid sodium salt (SA) (CAS Number: 9005-38-3, M/G ratio of about 1.56, molecular weight 280,000 in g/mol). Agar and sodium caseinate (CA) were provided by Grammol (Zagreb, Croatia). Commercial CaCl_2_ was purchased from Kemika (Zagreb, Croatia) and Eosin Y from Sigma Aldrich (USA). None of the other chemicals required additional purification; they were all analytical grade and utilized exactly as received.

#### 4.1.1. Bifidobacterium animalis subsp. lactis BB-12 Cells and Preparation for Encapsulation

The culture of *Bifidobacterium animalis* subsp. *lactis* BB-12^®^ (BB-12) obtained from Chr. Hansen (Hǿnsholm, Denmark) was freeze-dried, activated, and homogenized at 37 °C for 2 h. Moreover, 1 g/100 mL of BB-12 cells gave working biomass (9–9.5 log CFU/mL) for the process of encapsulation.

#### 4.1.2. Microparticles Preparation

Microparticles were prepared in one step by ionic gelation at room temperature in a sterile environment as described by Jurić et al. [42]. Two sets of microparticles were prepared: control microparticles, calcium alginate (Sample 0) and calcium alginate/casein (Sample 1), and composites with encapsulated BB-12, calcium alginate/agar/BB-12 (Sample 2) and calcium alginate/agar/casein/BB-12 (Sample 3). In brief, control microparticles were prepared by dripping 100 mL of SA (2% *w*/*v*) or a mixture of SA (2% *w*/*v*)/CA (0.5% *w*/*v*) into 100 mL of calcium chloride solution (3% *w*/*v*). Composites with encapsulated BB-12 were prepared as follows: freeze-dried BB-12 cells (1 g) were immediately added to 100 mL of SA (2% *w*/*v*)/agar (0.1% *w*/*v*) or SA (2% *w*/*v*)/agar (0.1% *w*/*v*)/CA (0.5 *w*/*v*%) mixtures and homogenized by slight mixing for 10 min (Biosan Orbital Shaker-Incubator ES-20, Medical-Biological Research & Technologies, Riga, Latvia). The mixture was dripped into 100 mL of CaCl_2_ (3% *w*/*v*) solution.

An encapsulator nozzle (750 μm) at 600 Hz vibration frequency and 121 mbar pressure (Encapsulator Büchi-B390 Bütchi Labortechnik AG, Flawil, Sankt Gallen, Switzerland) with steady magnetic stirring was used. To encourage gel strengthening, the produced microparticles were left at the room temperature for an additional 30 min. To eliminate excess CaCl_2_, microparticles were washed three times with sterile distilled water, filtered through a Büchner funnel, and kept at 4 °C for future investigations.

### 4.2. Methods

#### 4.2.1. The Zeta Potential and Size of BB-12 Cells Suspended in Water and Calcium Ions Solutions

Zetasizer Ultra (Malvern Panalytical, London, UK) with a 632.8 nm He-Ne laser was used to perform dynamic light scattering (DLS) measurements to determine the hydrodynamic diameter of nanoparticles suspended in water and calcium ions solutions (concentration ranged from 1 to 4% *w*/*v*). Samples were analyzed at three separate scattering angles (front 13°, side 90°, and back 173°) using the Multi-Angle Dynamic Light Scattering (MADLS^®^, Netanya, Israel) technology and the results were combined into a single integrated measurement. The measurements were carried out using DTS0012 1 cm plastic cuvettes. Hydrodynamic diameters were estimated using the number distributions, and the findings were provided as the averages of 3–5 measurements. Zeta potential measurements were carried out on the same equipment utilizing electrophoretic light scattering in DTS1070 folded capillary cells. The zeta potential values are expressed as the average of three measurements.

#### 4.2.2. Microscopic Observations

BB-12 cells and microparticles were analyzed by several microscopic techniques: (i) optical microscope (OM) (Leica MZ16a stereomicroscope, Leica Microsystems Ltd., Balgach, Switzerland), (ii) scanning electron microscope (SEM) (FE-SEM, model JSM-7000 F, Jeol Ltd., Akishima, Japan), and (iii) atomic force microscope (AFM) (Nanosurf CoreAFM, Nanosurf AG, Liestal, Switzerland). The average diameter of wet and dry composite microparticles was determined by optical microscopy using Olympus Soft Imaging Solutions GmbH, version E_LCmicro_09Okt2009. Forty composite microparticle formulations were randomly selected from batches produced in triplicate to determine the size distribution. The BB-12 size of colonies and cells shape were determined by optical microscopy using Olympus Soft Imaging Solutions GmbH, version E_LCmicro_09Okt2009. Dried samples for SEM analysis were put on highly conductive graphite tape. The FE-SEM was linked to an EDS/INCA 350 (energy dispersive X-ray analyzer) developed by Oxford Instruments Ltd. (Oxon, UK). ImageJ (Phyris software 9.1.0.0198) was used to determine the size of pores on a microparticle surface. The AFM images were acquired with the Nanosurf CoreAFM under ambient conditions. The non-contact mode was used for the acquisition with a 55% nominal value using a Tap300Al-G tip with a nominal spring constant of 40 N/m, a tip radius of less than 10 nm and a notional resonant frequency of 300 kHz on a 10 × 10 μm surface. The acquisition time was 0.78 s. The images were processed with the Nanosurf software 3.10.5. The surface roughness (Rq) is calculated by squaring each Z value (height) of the sample and taking the arithmetic mean of these values. In this way, the roughness is determined as the arithmetic average of the absolute heights over the entire height line of the sample so that the presence of a few major deviations can influence the roughness. The surface roughness was calculated at three different locations on the samples to increase accuracy. The images were processed using Gwyddion v1.4 software [83].

#### 4.2.3. Attenuated Total Reflectance Fourier Transform Infrared Spectroscopy

Fourier transform infrared spectroscopy in conjunction with the attenuated total reflectance (ATR-FTIR) recording technique was used to assess each sample. A Cary 660 FTIR spectrometer (Agilent Technologies, Palo Alto, CA, USA) and the Golden Gate single-reflection diamond ATR accessory (Specac) were used to obtain the materials’ FTIR-ATR spectra. The mid-infrared region (spectral range: 4000–400 cm^−1^) and transmission mode were used to record the spectra.

#### 4.2.4. Encapsulation Efficiency, Loading Capacity, Swelling Degree and In Vitro BB-12 Release from Microspheres

Detailed procedures for the determination of encapsulation efficiency (EE), loading capacity (EC), swelling degree (S_w_) and the fraction of released BB-12 (f_BB-12_) from alginate composites were previously described [84].

(a) Encapsulation efficiency

The encapsulation efficiency is the concentration of the encapsulated material detected in a microparticle over the initial concentration used to make the microparticle. The following formula was used to compute the encapsulation efficiency, which was given as a percentage of the live cells employed in the encapsulation [85]:

EE (%) = (N_com_/N_sus_) × 100 (4)

where N_com_ (expressed as the number of colony-forming unit (CFU) per gram of microparticles) and N_sus_ (expressed as CFU per gram of solution) represent viable counts in composites and cell suspension used for encapsulation, respectively. Original cell suspension used for encapsulation was serially diluted (1:10) in sterile saline solution (0.85% NaCl, Merck, Germany), plated on MRS agar (Biolife, Milan, Italy) plates in duplicates and incubated at 30 °C for 48 h under anaerobic conditions.

(b) Loading capacity

The loading capacity of a microparticle is the amount of encapsulated material loaded per unit weight. The loading capacity was determined by dissolving of 1 g of dry microparticles in 10 mL of a mixture of 0.2 mol/dm^3^ NaHCO_3_ + 0.06 mol/dm^−3^ Na_3_C_6_H_5_O_7_ × 2H_2_O [84,86]. The resulting solution was filtered and the number of cells was determined spectrophotometrically (Shimadzu 1900, Kyoto, Japan) at λ = 600 nm using the method of Waghunde et al. [87]. The loading capacity expressed as the number of BB-12 cells per 1 g of dry microparticles (CFU/g) was calculated by the following equation:

LC = c(CFU)/w(5)

where c(CFU) is a colony-forming unit and w is the weight of microparticles.

(c) Swelling degree

The swelling degree refers to the extent to which alginate hydrogels absorb water and swell when they come into contact with an aqueous environment. The swelling degree was calculated using the following equation:

(6) $$S_{w}=\left(\frac{w_{t}-w_{0}}{w_{0}}\right)\times 100$$

where w_t_ is the weight of the swollen microparticles and w_0_ is their initial weight.

(d) In vitro release of BB-12 from composites

Studies on the release of BB-12 cells from composites were carried out at room temperature. Composites (10 g) loaded with BB-12 cells were dispersed in 100 mL of deionized water and allowed to stand undisturbed during the experiments. At appropriate time intervals, the dispersion was mixed for 60 s, aliquots were taken and the number of cells was determined spectrophotometrically (Shimadzu 1900, Kyoto, Japan) at λ = 600 nm. The results are presented as the fraction of BB-12 cells released using the following equation:

f_BB-12_ = R_t_/R_tot_ (7)

where f_BB-12_ represents the fraction of BB-12 cells released, R_t_ is the amount of BB-12 cells released at time t, and R_tot_ is the total amount of BB-12 cells in the composite microparticles.

Experiments were conducted in triplicate at room temperature. IBM SPSS Statistics 22 and Microsoft Excel 2016 with the XLSTAT statistical software add-in were used to evaluate the collected data. The mean value and standard deviation were displayed for every data point.

#### 4.2.5. Rheological Measurements

The rheological characteristics of the microparticles were analyzed through oscillatory rheology. The storage (G′) and loss (G″) moduli were determined using a mechanical rheometer (Anton Paar MCR 302, Stuttgart, Germany) with a sandblasted steel plate−plate geometry (PP25/S, Anton Paar, Graz-Austria) and a gap of 1.5 mm. The instrument was equipped with a true-gap system, and data were collected using RheoCompass software (version 1.30). Adhesive tape was attached to the surface of the base plate to prevent the microparticles from slipping during the measurements. Sample temperature was regulated using Peltier temperature control located at the base of the geometry, along with a Peltier-controlled hood (H-PTD 200, Anton Paar, Stuttgart, Germany). During the experiment, a sample was positioned on the rheometer’s base plate, and the plate was adjusted using the true-gap function of the software. After 2 min at 23 °C, measurements of G′ and G″ moduli were consistently taken within the linear viscoelastic region (LVR). The yield stress was determined by conducting a strain (γ) sweep between 0.01% and 100% at a constant frequency of 5 rad/s. The rheological properties of the sample were found to be strain-independent up to the yield strain. Beyond the yield strain, the rheological behavior became nonlinear. Subsequent frequency sweeps (0.1–100 rad/s) were carried out at 23 °C, maintaining a strain value of 0.1% within the LVR to explore the time-dependent deformation behavior of the sample. Creep recovery experiments were conducted by subjecting samples to a shear stress (σ) of 30 Pa, a stress value determined to be within the linear viscoelastic region (LVR) for all specimens. This stress was applied for 5 min, during which strain measurements were recorded. Subsequently, the applied force was removed, and strain measurements were continued for an additional 10 min to observe the recovery stage. The creep was interpreted using the Burger model, which was successfully applied to describe the viscoelastic behavior of polymers.

#### 4.2.6. Differential Scanning Calorimetry

The thermal properties of dried samples were investigated with differential scanning calorimetry (DSC) using a PerkinElmer Diamond calorimeter operating in dynamic mode and calibrated with In and Zn standards. Samples (5–11 mg) were sealed into aluminum pans and heating and cooling cycles were performed for each sample with temperatures ranging from 25 °C to 350 °C in a pure nitrogen environment with a rate of 10 °C/min. For each sample, two–three specimens were recorded. The glass transition temperatures and the crystallization temperatures and enthalpies of samples were determined from the heating cycles.

## Figures and Tables

**Table 1:** Roughness parameters, average roughness (Ra), root mean square of roughness (Rq) and Z range of analyzed samples.

Sample	Ra/nm	Rq/nm	Zrange/nm
Sample 0	74 ± 6	90 ± 7	590 ± 39
Sample 1	84 ± 6	106 ± 9	655 ± 71
Sample 2	59 ± 3	71 ± 7	387 ± 41
Sample 3	76 ± 6	97 ± 8	657 ± 69

**Table 2:** Results of amplitude sweep tests of Sample 0, Sample 1, Sample 2 and Sample 3 examined at 23 °C.

	G′ (max)/Pa	Yield Point/ Stress/Pa	Yield Point/ Strain/%	Flow Point/Pa	Loss Factor	Flow Transition Index
Sample 0	17,524	240.1	1.54%	461.2	0.21	1.92
Sample 1	15,942	173.9	1.25%	371.1	0.20	2.13
Sample 2	35,700	377.8	1.38%	584.3	0.17	1.55
Sample 3	25,052	281.9	1.34%	482.1	0.18	1.71

**Table 3:** γ_max_, J_max_, J_0_, η_0_, J_e0_, elastic compliance J_e_ (%) for Samples 0–4 were determined at 25 °C.

Creep/Creep Recovery	Max. Strain γ_max_ (%)	Max. Creep Compliance J_max_ (1/Pa) × 10^−4^	Instantaneous Compliance J_0_ (1/Pa) × 10^−4^	Zero Shear Viscosity η_0_ (mPa·s) × 10^9^	Compliance J_e0_ (1/Pa) × 10^−5^	Elastic Compliance J_e_ (%)
Sample 0	0.83	2.78	1.38	8.93	9.80	35.3
Sample 1	0.80	2.64	1.24	8.59	8.58	32.4
Sample 2	0.57	1.90	0.94	13.03	7.99	41.9
Sample 3	0.66	2.22	1.15	14.14	8.00	36.2

**Table 4:** Transition temperatures and corresponding enthalpies (ΔH) of Sample 0, Sample 1, Sample 2 and Sample 3 from the first heating cycle at a heating rate of 10 °C/min: T_endo_ (the first endothermic transition), T_g_ (the glass transition temperature) and T_cc_ (the cold crystallization temperature).

Samples	First Transition	Second Transition	Third Transition
Sample 0	T_endo_ = 91 °C	Tg = 192.63 °C	Tcc = 294.36 °C
		ΔCp = 7.97 J/g·°C	ΔHcc = −106 J/g
		ΔH = 39.73 J/g	
Sample 1	T_endo_ = 111 °C	Tg = 211.93 °C	Tcc = 288.08 °C
		ΔCp = 7.85 J/g·°C	ΔHcc = −73.51 J/g
		ΔH = 34.13 J/g	
Sample 2	T_endo_ = 108 °C	Tg = 196.02 °C	Tcc = 291.08 °C
		ΔCp = 10.96 J/g·°C	ΔHcc = −54.55 J/g
		ΔH = 20.99 J/g	
Sample 3	T_endo_ = 115 °C	Tg = 200.75 °C	Tcc = 290.22 °C
		ΔCp = 11.73 J/g·°C	ΔHcc = −52.15 J/g
		ΔH = 20.73 J/g	

## Data Availability

The data presented in this study are available on request from the corresponding authors.

## References

[1] Bermudez-Brito M., Plaza-Díaz J., Muñoz-Quezada S., Gómez-Llorente C., Gil A. (2012). Probiotic mechanisms of action. Ann. Nutr. Metab..

[2] Sanders M.E. (2003). Probiotics: Considerations for human health. Nutr. Rev..

[3] Cook M.T., Tzortzis G., Charalampopoulos D., Khutoryanskiy V.V. (2012). Microencapsulation of probiotics for gastrointestinal delivery. J. Control. Release.

[4] Sbehatm M., Mauriello G., Altamimi M. (2022). Microencapsulation of Probiotics for Food Functionalization: An Update on Literature Reviews. Microorganisms.

[5] Shori A.B. (2017). Microencapsulation Improved Probiotics Survival During Gastric Transit. HAYATI J. Biosci..

[6] Draget K.I., Skjåk-Bræk G., Smidsrød O. (1994). Alginate based new materials. Carbohydr. Polym..

[7] Ching S.H., Bansal N., Bhandari B. (2017). Alginate gel particles-A review of production techniques and physical properties. Crit. Rev. Food Sci. Nutr..

[8] Kwiecień I., Kwiecień M. (2018). Application of Polysaccharide-Based Hydrogels as Probiotic Delivery Systems. Gels.

[9] George M., Abraham T.E. (2006). Polyionic hydrocolloids for the intestinal delivery of protein drugs: Alginate and chitosan—A review. J. Control. Release.

[10] Agriopoulou S., Tarapoulouzi M., Varzakas T., Jafari S.M. (2023). Application of Encapsulation Strategies for Probiotics: From Individual Loading to Co-Encapsulation. Microorganisms.

[11] Guérin D., Vuillemard J.-C., Subirade M. (2003). Protection of Bifidobacteria Encapsulated in Polysaccharide-Protein Gel Beads against Gastric Juice and Bile. J. Food Prot..

[12] Khlibsuwan R., Khunkitti W., Pongjanyakul T. (2018). Alginate-caseinate composites: Molecular interactions and characterization of cross-linked beads for the delivery of anticandidals. Int. J. Biol. Macromol..

[13] Wang X., Gao S., Yun S., Zhang M., Peng L., Li Y., Zhou Y. (2022). Microencapsulating Alginate-Based Polymers for Probiotics Delivery Systems and Their Application. Pharmaceuticals.

[14] Ramdhan T., Ching S.H., Prakash S., Bhandari B. (2020). Physical and mechanical properties of alginate based composite gels. Trends Food Sci. Technol..

[15] Frakolaki G., Giannou V., Tzia C. (2023). Encapsulation of Bifidobacterium animalis subsp. lactis Through Emulsification Coupled with External Gelation for the Development of Synbiotic Systems. Probiotics Antimicrob. Proteins.

[16] Yin Z.-C., Wang Y.-L., Wang K. (2018). A pH-responsive composite hydrogel beads based on agar and alginat for oral drug delivery. J. Drug Deliv..

[17] Mostafavi F.S., Zaeim D. (2020). Agar-based edible films for food packaging applications—A review. Int. J. Biol. Macromol..

[18] Ghazagh P., Frounchi M. (2024). Hydroxyapatite/alginate/polyvinyl alcohol/agar composite double-network hydrogels as injectable drug delivery microspheres. Chem. Pap..

[19] Ghosh A.K., Bandyopadhyay P., Karunaratne D.N. (2012). Polysaccharide-protein interactions and their relevance in food colloids. The Complex World of Polysaccharides.

[20] Phadungath C. (2005). Casein micelle structure: A concise review. Songklanakarin. J. Sci. Technol. (SJST).

[21] He Z., Zhang X., Qi W., Qui W., Guang R., Su E. (2015). Alginate-casein microspheres as bioactive vehicles for nutrients. Trans. Tianjin Univ..

[22] Yang X., Li A., Li D., Guo Y., Sun L. (2021). Applications of mixed polysaccharide-protein systems in fabricating multi-structures of binary food gels—A review. Trends Food Sci. Technol..

[23] Chen L., Subirade M. (2009). Alginate-whey protein granular microspheres as oral delivery vehicles for bioactive compounds. Biomacromolecules.

[24] Bonnaillie L.M., Zhang H., Akkurt S., Yam K.L., Tomasula P.M. (2014). Casein Films: The Effects of Formulation, Environmental Conditions and the Addition of Citric Pectin on the Structure and Mechanical Properties. Polymers.

[25] Sadiq U., Gill H., Chandrapala J. (2021). Casein Micelles as an Emerging Delivery System for Bioactive Food Components. Foods.

[26] Li P., Guo C., Li X., Yuan K., Yang X., Guo Y., Yang X. (2021). Preparation and structural characteristics of composite alginate/casein emulsion gels: A microscopy and rheology study. Food Hydrocoll..

[27] Mojaveri S.J., Hosseini S.F., Gharsallaoui A. (2020). Viability improvement of Bifidobacterium animalis BB-12 by encapsulation in chitosan/poly(vinyl alcohol) hybrid electrospun fiber mats. Carbohydr. Polym..

[28] Loyeau P.A., Spotti M.J., Vanden Braber N.L., Rossi Y.E., Montenegro M.A., Vinderola G., Carrara C.R. (2018). Microencapsulation of Bifidobacterium animalis subsp. lactis INL1 using whey proteins and dextrans conjugates as wall materials. Food Hydrocoll..

[29] Holkem A.T., Raddatz G.C., Barin J.S., Moraes Flores É.M., Muller E.I., Codevilla C.F., Jacob-Lopes E., Grosso C.F.-G., de Menezes C.R. (2017). Production of microparticles containing Bifidobacterium BB-12 by emulsification/internal gelation. LWT.

[30] Frakolaki G., Giannou V., Kekos D., Tzia C. (2021). A review of the microencapsulation techniques for the incorporation of probiotic bacteria in functional foods. Crit. Rev. Food Sci. Nutr..

[31] Cedran M.F., Rodrigues F.J., Bicas J.L. (2021). Encapsulation of Bifidobacterium BB-12^®^ in alginate-jaboticaba peel blend increases encapsulation efficiency and bacterial survival under adverse conditions. Appl. Microbiol. Biotechnol..

[32] Rathore S., Desai P.M., Liew C.V., Chan L.W., Heng P.W.S. (2013). Microencapsulation of microbial cells. J. Food Eng..

[33] Arenas-Padilla M., Duarte-Gutiérrez J.L., Mata-Haro V. (2018). Bifidobacterium animalis ssp. lactis BB-12 induces IL-10 through cell membrane-associated components via TLR2 in swine. J. Appl. Microbiol..

[34] Milani C., Turroni F., Duranti S., Lugli G.-A., Mancabelli L., Ferrario C., van Sinderen D., Ventura M. (2015). Genomics of the Genus Bifidobacterium Reveals Species-Specific Adaptation to the Glycan-Rich Gut Environment. Appl. Environ. Microbiol..

[35] Fachin L., Moryia J., Neves Gândara A.L., Viotto W.H. (2008). Evaluation of culture media for counts of Bifidobacterium animalis subsp. lactis Bb 12in yoghurt after refrigerated storage. Braz. J. Microbiol..

[36] Indira M., Venkateswarulu T.C., Abraham Peele K., Nazneen Bobby M., Krupanidhi S. (2019). Bioactive molecules of probiotic bacteria and their mechanism of action: A review. 3 Biotech.

[37] Gibson G.R., Roberfroid M.B. (1995). Dietary modulation of the human colonic microbiota: Introducing the concept of prebiotics. J. Nutr..

[38] Dufrêne Y.F., Viljoen A., Mignolet J., Mathelié-Guinlet M. (2021). AFM in cellular and molecular microbiology. Cell. Microbiol..

[39] Dhanashree, Rajashekharan S., Krishnaswamy B., Kammara R. (2017). Bifid Shape Is Intrinsic to Bifidobacterium adolescentis. Front. Microbiol..

[40] Bozkurt K., Denktas C., Ozdemir O., Altındal A., Avdan Z., Bozkurt H.S. (2019). Charge Transport in Bifidobacterium animalis subsp. Lactis BB-12 under Various Atmospheres. Open J. Appl. Sci..

[41] Milani C., Lugli G.A., Duranti S., Turroni F., Bottacini F., Mangifesta M., Sanchez B., Viappiani A., Mancabelli L., Taminiau B. (2014). Genomic encyclopedia of type strains of the genus Bifidobacterium. Appl. Environ. Microbiol..

[42] Jurić S., Tanuwidjaja I., Fuka M.M., Kahlina K.V., Marijan M., Boras A., Vinceković M. (2020). Encapsulation of two fermentation agents, Lactobacillus sakei and calcium ions in mirospheres. Colloids Surf. B.

[43] Oust A., Møretrø T., Kirschner C., Narvhus J.A., Kohler A. (2004). FT-IR spectroscopy for identification of closely related lactobacilli. J. Microbiol. Methods.

[44] Shakirova L., Auzina L., Zikmanis P., Gavare M., Grube M. (2010). Influence of Growth Conditions on Hydrophobicity of Lactobacillus acidophilus and Bifidobacterium lactis Cells and Characteristics by FT-IR Spectra. Spectroscopy.

[45] Sharma P.K., Das A., Rao K.H., Forssberg K.S.E. (2003). Surface Characterization of Acidithiobacillus ferrooxidans Cells Grown under Different Conditions. Hydrometallurgy.

[46] Vinceković M., Šegota S., Jurić S., Harja M., Ondrašek G. (2022). Development and Characterization of a Novel Soil Amendment Based on Biomass Fly Ash Encapsulated in Calcium Alginate Microspheres. Int. J. Mol. Sci..

[47] Arfat Y.A., Ahmed J., Jacob H. (2017). Preparation and characterization of agar-based nanocomposite films reinforced with bimetallic (Ag-Cu) alloy nanoparticles. Carbohydr. Polym..

[48] Mahmoud Nasef M., El-Hefian E.A., Saalah S., Yahaya A.H. (2011). Preparation and Properties of Non-Crosslinked and Ionically Crosslinked Chitosan/Agar Blended Hydrogel Films. E-J. Chem..

[49] Nagaraju P.G., Sindhu P., Dubey T., Chinnathambi S., Poornima Priyadarshini C.G., Rao P.J. (2021). Influence of sodium caseinate, maltodextrin, pectin and their Maillard conjugate on the stability, in vitro release, anti-oxidant property and cell viability of eugenol-olive oil nanoemulsions. Int. J. Biolog. Macromol..

[50] Barreto P.L.M., Pires A.T.N., Soldi V. (2003). Thermal degradation of edible films based on milk proteins and gelatin in inert atmosphere. Polym. Degrad. Stab..

[51] Jurić S., Đermić E., Topolovec-Pintarić S., Bedek M., Vinceković M. (2019). đ Physicochemical properties and release characteristics of calcium alginate microspheres loaded with Trichoderma viride spores. J. Integr. Agric..

[52] Malektaj H., Drozdov A.D., de Claville Christiansen J. (2023). Mechanical Properties of Alginate Hydrogels Cross-Linked with Multivalent Cations. Polymers.

[53] Abarca-Cabrera L., Fraga-García P., Berensmeier S. (2021). Bio-nano interactions: Binding proteins, polysaccharides, lipids and nucleic acids onto magnetic nanoparticles. Biomater. Res..

[54] Sreya E.S., Kumar D.P., Balakrishnan P., Gopi S., Thomas S., Hosur M., Pasquini D., Jose Chirayil C. (2023). Science and Technology of Alginates: A Review. Handbook of Biomass.

[55] McMahon D.J., Oommen B.S., McSweeney P., Fox P. (2013). Casein Micelle Structure, Functions, and Interactions. Advanced Dairy Chemistry.

[56] Zhang X., Wang K., Hu J., Zhang Y., Dai Y., Xia F. (2020). Role of a high calcium ion content in extending the properties of alginate dual-crosslinked hydrogels. J. Mater. Chem. A.

[57] Paoletti S., Donati I. (2022). Comparative Insights into the Fundamental Steps Underlying Gelation of Plant and Algal Ionic Polysaccharides: Pectate and Alginate. Gels.

[58] Elzoghby A.O., El-Fotoh W.S., Elgindy N.A. (2011). Casein-based formulations as promising controlled release drug delivery systems. J. Control. Release.

[59] Bennacef C., Desobry S., Jasniewski J., Leclerc S., Probst L., Desobry-Banon S. (2023). Influence of Alginate Properties and Calcium Chloride Concentration on Alginate Bead Reticulation and Size: A Phenomenological Approach. Polymers.

[60] Wang Y., Lu Y. (2023). Sodium Alginate-Based Functional Materials toward Sustainable Applications: Water Treatment and Energy Storage. Ind. Eng. Chem. Res..

[61] Jurić S., Šegota S., Vinceković M. (2019). Influence of surface morphology and structure of alginate microparticles on the bioactive agents release behavior. Carbohydr. Polym..

[62] Vinceković M., Jurić S., Vlahoviček-Kahlina K., Martinko K., Šegota S., Marijan M., Krčelić A., Svečnjak L., Majdak M., Nemet I. (2023). Novel Zinc/Silver Ions-Loaded Alginate/Chitosan Microparticles Antifungal Activity against Botrytis cinerea. Polymers.

[63] Jabeen S., Maswal M., Chat O.A., Rather G.M., Dar A.A. (2016). Rheological behavior and Ibuprofen delivery applications of pH responsive composite alginate hydrogels. Colloids Surf. B Biointerfaces.

[64] Cuomo F., Cofelice M., Lopez F. (2019). Rheological characterization of hydrogels from alginate-based nanodispersion. Polymers.

[65] Wang J., Chen Z., Zhang W., Lei C., Li J., Hu X., Zhanf F., Chen C. (2023). The physical and structural properties of acid-Ca2+ induced casein-alginate/Ca2+ double network gels. Int. J. Biol. Macromol..

[66] Dodero A., Vicini S., Alloisio M., Castellano M. (2019). Sodium alginate solutions: Correlation between rheological properties and spinnability. J. Mater. Sci..

[67] Olderøy M.Ø., Xie M., Andreassen J.P., Strand B.L., Zhang Z., Sikorski P. (2012). Viscoelastic properties of mineralized grains of alginate hydrogel. J. Mater. Sci. Mater. Med..

[68] Huang D., Quan Q., Zheng Y., Tang W., Zhang Z., Qiang X. (2020). Dual-network design to enhance the properties of agar aerogel adsorbent by incorporating in situ ion cross-linked alginate. Environ. Chem. Lett..

[69] Leick S., Kott M., Degen P., Henning S., Päsler T., Suter D., Rehage H. (2011). Mechanical properties of liquid-filled shellac composite capsules. Phys. Chem. Chem. Phys. PCCP.

[70] Stephen A.M., Phillips G.O. (2006). Food Polysaccharides and Their Applications.

[71] Steffe J.F.R. (1996). Rheological Methods in Food Process Engineering.

[72] Bellich B., Borgogna M., Cok M., Cesàro A. (2011). Release Properties of Hydrogels: Water Evaporation from Alginate Gel Beads. Food Biophys..

[73] Vinceković M., Jurić S., Šegota S., Šijaković Vujičić N., Španić N., Mutaliyeva B., Prosyanik A.V., Marijan M. (2022). Morphological, rheological and thermal characteristics of biopolymeric microparticles loaded with plant stimulants. J. Polym. Res..

[74] Florea-Sprioiu A., Bala D., Balan A., Nichita C., Stamatin I. (2012). Alginate matrices prepared in sub and supercritical CO_2_. Dig. J. Nanomater. Biostruct..

[75] E Hariri El Nokab M., Es Sayed J., De Witte F., Dewettinck K., Elshewy A., Zhang Z., Van Steenberge P.H.M., Wang T., Sebakhy K.O. (2024). A comparative analytical study for the different water pools present in alginate hydrogels: Qualitative vs. quantitative approaches. Food Hydrocoll..

[76] Bagre A.C., Jain K., Jain N.K. (2013). Alginate coated chitosan coreshell nanoparticles for oral delivery of enoxaparin: In vitro and in vivo assessment. Int. J. Pharm..

[77] Riberio A.J., Silva C., Ferreira D., Vega F. (2005). Chitosan-reinforced alginate microspheres obtained through the emulsification/internal gelation technique. Eur. J. Pharm. Sci..

[78] Soares J.P., Santos J.E., Chierice G.O., Cavalheiro E.T.G. (2004). Thermal behavior of alginic acid and its sodium salt. Eclética Química.

[79] Gargallo L., Radić D. (2009). Viscoelastic Behaviour of Polymers. Physicochemical Behavior and Supramolecular Organization of Polymers.

[80] Siepmann J., Siepmann F. (2012). Modeling of diffusion controlled drug delivery. J. Control. Release.

[81] Korsmeyer R.W., Gurny R., Doelker E., Buri P., Peppas N.A. (1983). Mechanisms of solute release from porous hydrophilic polymers. Int. J. Pharm..

[82] Kim H., Fassihi R. (1997). Application of a binary polymer system in drug release rate modulation. 2. Influence of formulation variables and hydrodynamic conditions on release kinetics. J. Pharm. Sci..

[83] Nečas D., Klapetek P. (2012). Gwyddion: An open-source software for SPM data analysis. Cent. Eur. J. Phys..

[84] Vinceković M., Jalšenjak N., Topolovec-Pintarić S., Đermić E., Bujan M., Jurić S. (2016). Encapsulation of biological and chemical agents for plant nutrition and protection: Chitosan/Alginate microcapsules loaded with copper cations and Trichoderma viride. J. Agric. Food Chem..

[85] Corbo M.R., Bevilacqua A., Speranza B., Di Maggio B., Gallo M., Sinigaglia M. (2016). Use of alginate beads as carriers for lactic acid bacteria in a structured system and preliminary validation in a meat product. Meat Sci..

[86] Vinceković M., Jurić S., Đermić E., Topolovec-Pintarić S. (2017). Kinetics and mechanisms of chemical and biological agents release from biopolymeric microcapsules. J. Agric. Food Chem..

[87] Waghunde R.R., Priya J., Naik B.M., Solanky K.U., Sabalpara A.N. (2010). Optical density—A tool for the estimation of spore count of Trichoderma Viride. J. Biopestic..

